# The role of Notch signalling in ovarian angiogenesis

**DOI:** 10.1186/s13048-017-0308-5

**Published:** 2017-03-11

**Authors:** Qi Xie, Zuowang Cheng, Xiaocui Chen, Corrinne G. Lobe, Ju Liu

**Affiliations:** 1 Laboratory of Microvascular Medicine, Medical Research Center, Shandong Provincial Qianfoshan Hospital, Shandong University, 16766 Jingshi Road, Jinan, People’s Republic of China; 2 Taishan Medical College, Taian, People’s Republic of China; 3 Molecular and Cellular Biology Division, Sunnybrook Health Science Centre, University of Toronto, Toronto, ON Canada; 4 Department of Medical Biophysics, University of Toronto, Toronto, ON Canada

**Keywords:** Notch signalling pathway, Angiogenesis, Ovarian cancer, Polycystic ovary syndrome, Dll4, Jagged 1, VEGF, Nitric Oxide

## Abstract

In adults, the ovary is characterized with extensive angiogenesis and regular intervals of rapid growth. Ovarian function is dependent on the network of angiogenic vessels which enable the follicle and/or corpus luteum to receive oxygen, nutrients and hormonal support. Abnormal angiogenesis is involved in the induction and development of pathological ovary, such as polycystic ovary syndrome and ovarian cancer. Notch signalling pathway is one of the primary regulators of angiogenesis and a therapeutic target for ovarian diseases. Here, we will review literatures on the expression pattern of Notch pathway components in the ovary and on the role of Notch signalling pathway on ovarian angiogenesis.

## Main text

Angiogenesis is the complex process by which new blood vessels develop from existing vessels. Vascular endothelial growth factor (VEGF) and its receptors are critical in angiogenesis. It is demonstrated that VEGF was from various sources. Especially, VEGF expression was higher in malignant tissues where VEGF is mainly secreted by tumor cells. VEGF gene expression is regulated by hypoxia, growth factor and hormones under physiological and pathological process, such as wounding healing, the female reproductive cycle or tumorigenesis. The main player in the response to VEGF is the endothelial cell. In response to pro-angiogenic stimulus, capillaries undergo a series of processes, including degradation of the extracellular matrix, endothelial cell proliferation and migration. At the apex of the sprout, endothelial cells differentiate into tip cells which are characterized with highly motile, tubeless, nonproliferative phenotypes. However, stalk cells adjacent to tip cells are highly proliferative. The tip cells extend numerous filopodia in reaction to stimulus, leading the direction of the new sprout while the stalk cells form the trunk of the new blood vessel. The vascular homeostasis is highly regulated by Notch signalling. Notch receptors mediate endothelial cell differentiation between tip or tube phenotypes [1]. Upon activation of Notch signalling, stalk cells inhibit excess sprout formation through down-regulation of expressions of VEGF receptors. Notch ligands Dll4 and Jagged 1 function oppositely in regulating angiogenesis. In adults, ovary is one of the few organs which maintain normal physiology by angiogenesis. Abnormal angiogenesis is involved in pathogenesis of ovarian diseases. This review will summarize the role of Notch signalling pathway in angiogenesis at both normal and pathological conditions.

## Angiogenesis in the normal and pathological ovary

### Angiogenesis in the normal ovary

The menstrual cycle can be divided into three phases in the ovary: follicular phase, ovulation and luteal phase. Follicles in the ovary develop under the effects of hormones. After several days, one or occasionally two become dominant follicles while non-dominant follicles shrink. By stimulation with the luteinizing hormone, the dominant follicle releases an oocyte, and the remains of the follicle becomes a corpus luteum (CL) which produces progesterone for pregnancy. Ovarian function is dependent on the establishment and continuous remodelling of vascular system which enables the follicles and CLs to receive the required supply of nutrients, oxygen and hormonal support [2]. Before ovulation, primordial and primary follicle mainly rely on support from stromal blood vessels. Capillaries grow into the follicle membrane layer after the development of primordial follicle. The primordial follicle then develops to preantral follicle and antral follicle with increasing microvascular density. Eventually, a new vascular bed forms in the process of follicular development [2]. Angiogenesis inhibition leads to the attenuation of follicular growth, disruption of ovulation and drastic effects on development of the CL [2]. Thus, increased thecal vascularity is required for maintaining follicular function, while reduced thecal vascularity is an important component of follicular atresia.

### Angiogenesis in polycystic ovary syndrome

Polycystic ovary syndrome (PCOS), a common endocrine disorder which impacts approximately 7% of women in reproductive age, is a leading cause of poor fertility [3]. The polycystic ovary is characterized by an increased stromal volume and more antral follicles localized around the periphery of the ovary. Thecal-stromal vascular density is increased in the ovary from PCOS patients compared with normal ovary [4]. PCOS also exhibits increased follicular vascularity and vascular permeability [5]. The increased vascularity of the ovary may contribute to the ovarian phenotype as disruption of the ovarian vasculature with diathermy leads to the follicular atresia and subsequent improvement of ovarian function [6]. Abnormal vascularization in the polycystic ovary may be attributed to the dysregulation of angiogenic factors in PCOS. The significant differences on the levels of VEGF, placental growth factor (PlGF), angiopoietins, transforming growth factor beta (TGF-β), platelet-derived growth factor (PDGF), and basic fibroblast growth factor (bFGF) in the ovary, follicular fluid, and the circulation were found between patients with PCOS and normal women, suggesting that multiple pro-angiogenic factors are involved in abnormal angiogenesis in PCOS [7].

### Angiogenesis in ovarian cancer

Ovarian cancer, one of the most common fatal gynecological malignancies, is the heterogeneous, rapidly progressive, and highly lethal disease. Expect ovarian cancers derived from other organs (metastatic cancers), ovarian tumors can be broadly classified into three categories, those derived from the surface epithelium, the germ cells and the specialized stroma. Tumors derived from the surface of the ovary is the most common form (about 90%) of ovarian cancer and occur primarily in adults. Further, the epithelial neoplasms are classified as serous (30–70%), endometrioid (10–20%), mucinous (5–20%), clear cell (3–10%), and undifferentiated (1%) [8]. Angiogenesis is a hallmark in ovarian cancer, and it is a key process that enables tumor growth and metastasis. Increased vascular density in ovarian cancer has been positively associated with an increased incidence of metastasis as well as decreased patient survival rates [9].

### The mechanisms of angiogenesis in normal ovary

The physiological angiogenesis during folliculogenesis, ovulation and luteal development requires the cooperation of multiple pro-angiogenic factors. R S Robinson etc. have proposed the mechanisms by which the CL is vascularised in primates [2]. In the pre-ovulatory follicle, the granulosa layer remains avascular, while extensive vascularisation is observed in the theca. VEGFA and FGF2 accumulate during follicular development. Proteolytic activity is increased following luteinizing hormone (LH) stimulation, and the basement membrane is degraded, leading to the release of angiogenic factors. The increase of VEGFA and FGF2 induces migration of endothelial cells to granulosa, endothelial proliferation and sprouting of existing vasculature. Later blood flow is reinitiated after tube formation and recruitment of pericytes. Lastly, angiogenic factors facilitate vessel stabilisation and maturation [2].

## Overview of the Notch signalling pathway

### Structure of Notch receptors

**Fig. 1:**
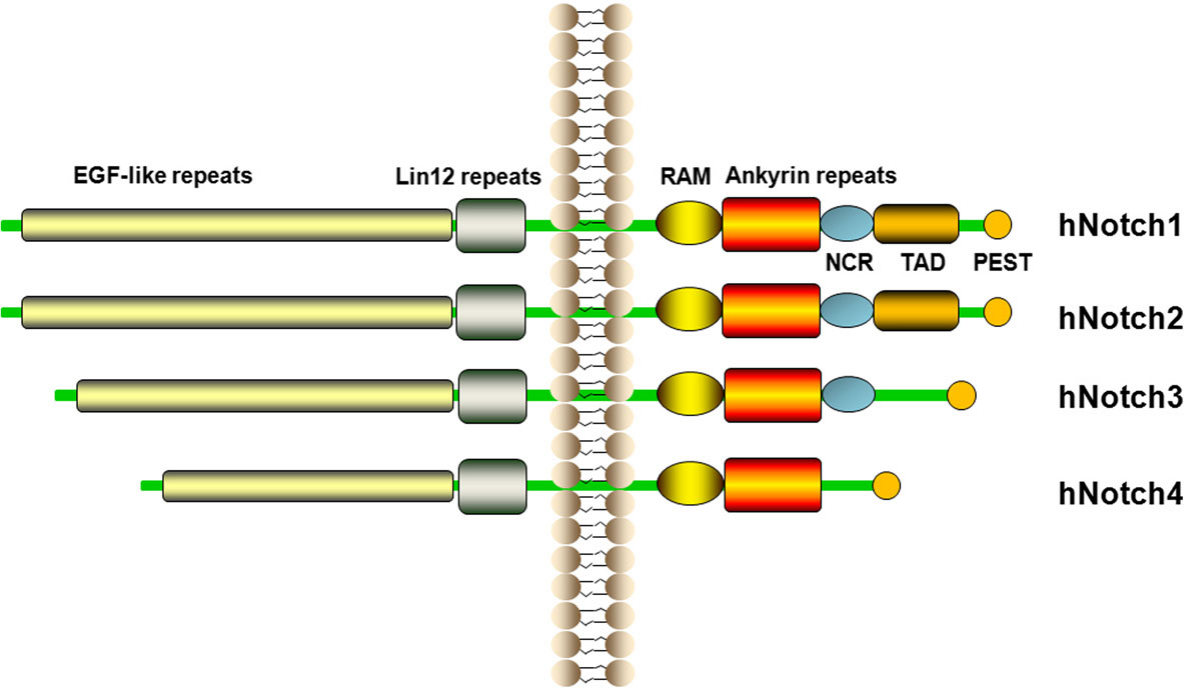
The four human receptors Notch1, 2, 3, 4. Human have four receptors: Notch1, 2, 3, 4. The Notch receptor has an extracellular domain consisting of EGF-like repeats with three cysteine-rich Lin12 repeats and intracellular domain containing a RAM23 domain, six ankyrin repeats, a transactivation domain (TAD) and a PEST sequence. Two nuclear localization sequences are present prior to and following the ankyrin repeats. Of note, Notch 1–3 contain cytokine response sequences (NCR) and The TAD is absent in Notch 3 and Notch 4

The Notch signalling pathway was originally discovered by Thomas Hunt Morgan through genetic studies in 1910 [10]. Mammals have 5 ligands (Jagged 1 2; Delta like 1, 3,4 and 4 receptors (Notch 1, 2, 3, 4). The Notch 1 protein is a 300kD single-pass receptor which has an extracellular domain containing of multiple epidermal growth factor (EGF)-like repeats with 3 cysteinerich Lin12 repeats and a heterodimerization domain (HD), a transmembrane domain (TD) and an intracellular domain containing a RAM23 domain, 6 ankyrin repeats, a transactivation domain (TAD) and a proline, glutamate, serine, threonine rich (PEST) sequence. Two nuclear localization sequences (NLS) located both sides of the ankyrin repeats. The TAD is absent in Notch 3 and Notch 4 protein (Fig. 1).

### Notch intracellular signal transduction

**Fig. 2:**
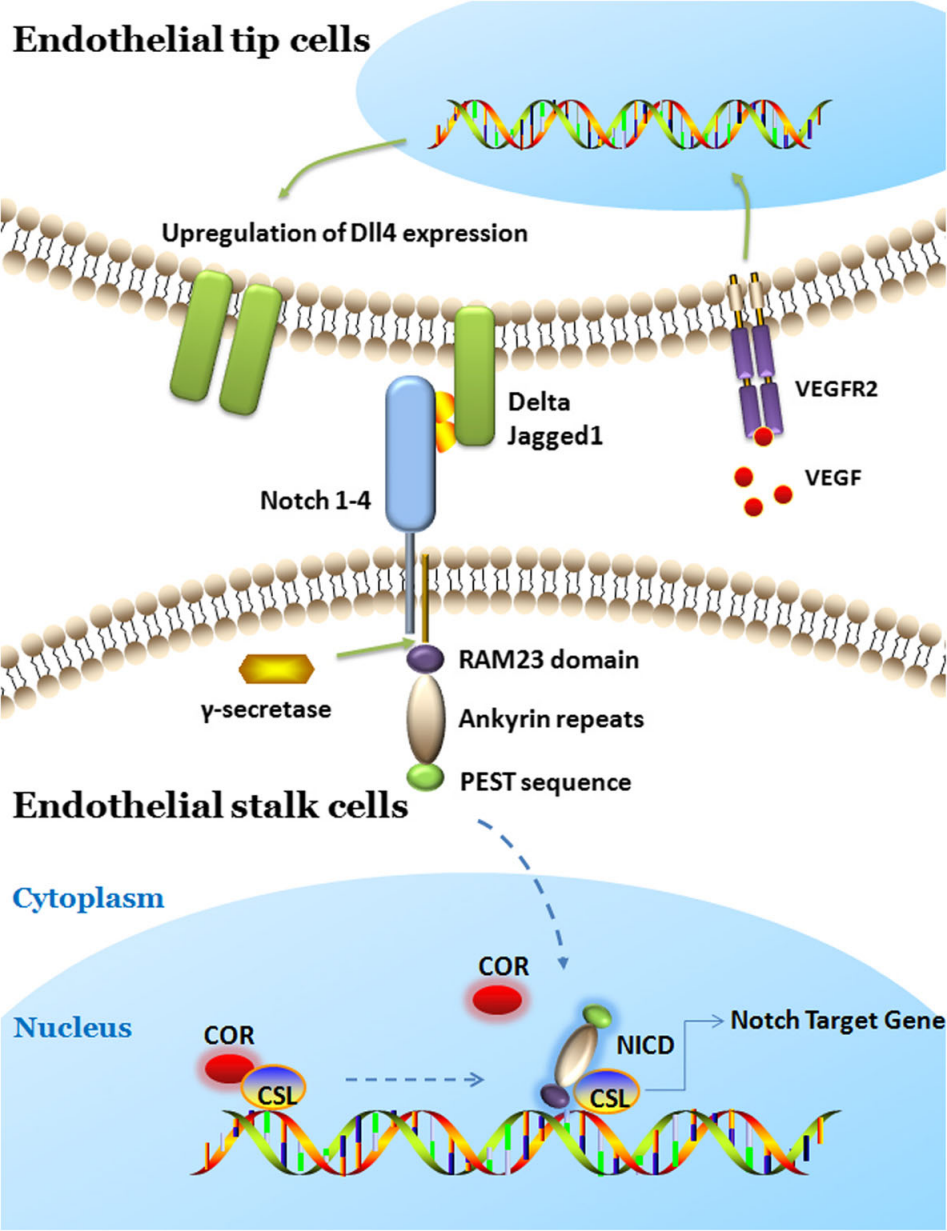
Targeting Notch signalling pathway regulates angiogenesis in the pathological ovary. Interaction of Notch receptors with Notch ligands, such as Delta-like or Jagged, between two endothelial tip cells and stalk cells leads to a cascade of proteolytic cleavages. Notch intracellular domain (NICD) is released from the cell membrane by γ-secretase complex. Then, NICD translocates to the nucleus, where it interacts with the DNA-binding protein RBP-Jkappa, thus further activating the transcription of Notch target genes. Further, endothelial cell are activated and network formation is induced during angiogenesis in pathological ovary. Different strategies have been used to block Notch signalling by using anti-Dll4 monoclonal antibodies, γ-secretase inhibitors, anti-Notch antibodies, or Notch1-trap. Given that crosstalking of VEGF and Notch play an important role in angiogenesis in pathological ovary, VEGF could be blocked by anti-VEGF antibodies

Notch activation involves proteolytic cleavages at 3 sites, leading to the release of the soluble intracellular domain of Notch (IC-Notch) from the membrane. Through the 2 NLS sites, IC-Notch translocates into the nucleus and then binds to CSL (CBF1/Suppressor of Hairless/Lag-1) via the RAM23 domain [11]. The CSL protein (also called CBF-1/RBP-J) binds to a specific DNA sequence GTGGGAA in the promoter region of Notch-regulated genes [12]. In the absence of IC-Notch, CSL forms a complex with SMRT (silencing mediator of retinoid and thyroid hormone receptors) and histone deacetylase (HDAC) to inhibit the transcription [13]. Binding of IC-Notch displaces HDAC and permits the recruitment of histone acetylases and the nuclear protein Mastermind, resulting in conversion of CSL from a repressor to an activator [14] (Fig. 2).

### Downstream targets of the Notch pathway

The best characterized target genes of Notch/CSL coactivator complexes are Hairy and Enhancer of Split (HES) family, which encode basic-helix-loop-helix (bHLH) transcription factors [15]. HES proteins inhibits the transcription of other bHLH proteins which are transcription activators, including MASH1 and MyoD [16, 17]. Transcriptional repression by the Hes proteins requires interaction with a co-repressor Groucho (also called TLE in human) [18]. Another related bHLH protein family, HERP (HES-related repressor protein, gridlock/Hey/Hesr/HRT), is also the target of Notch/CSL. HERPs share similar domains with Hes proteins and function as transcriptional repressors [19]. However, HERPs lack of binding motif for Groucho. HES and HERP may form a heterodimer and cooperate in transcriptional repression [19].

## The expressions of Notch pathway elements in the ovary

Notch proteins and ligands have been detected in the rodent ovary. Vorontchikhina et al. have characterized the expression patterns of Notch 1, Notch 4, and Jagged 1 proteins during the process of folliculogenesis and corpus luteum formation in the mouse ovary [20]. In the follicular phase ovary, Notch 1 was expressed within the endothelium of the theca layer. In the luteal phase ovary, the expression of Notch 1 was found in endothelial cells from neo-vasculature of corpora lutea and mature vessels of theca layer and Notch 1 expression in PECAM (platelet endothelial cell adhesion molecule) positive neovessels and mature vessels was maintained in the pregnancy phase in the stimulated ovaries. Notch 4 expression was found in PECAM positive endothelial cells in all stages of folliculogenesis and corpus luteum formation. Jagged 1 in the ovary was also found in both endothelial and αSMA positive mural cells in the unstimulated immature ovary. In the follicular phase ovary, Jagged 1 expression was found in the endothelial cells of the neovessels. And high level of Jagged 1 expression in the mature vasculature was maintained in the luteal and pregnancy phase. Also, Jagged 1 was expressed in the endothelial cells of the neovessels within the corpus lutea of luteal phase and pregnant ovaries.

In mouse, Joshua Johnson etc. have found that Notch 2, Notch 3, and Jagged 2 were expressed in the granulosa cells of developing follicles but Jagged 1 was expressed in oocytes exclusively [21]. Dll4 is primarily expressed on the endothelial cells at the tip of new vessels [22]. Jovanovic etc. also have determined the specific localization of Notch ligands and receptors: Dll4 is expressed in theca layer endothelial cells (ECs); Notch 1/Notch 4 and Jagged 1 are expressed in theca layer ECs and vascular smooth muscle cells (VSMCs), whereas Notch 3 is restricted to VSMCs; Notch 2 is expressed mostly on in granulose cells of small follicles [23].

Thus, Notch receptors and ligands are expressed in a subset of ovarian vessels, including both mature ovarian vasculature as well as angiogenic neovessels.

## The regulation of Notch pathway on angiogenesis in normal ovary

ECs express the Jagged 1, Dll1, and Dll4 ligands. Among the various Notch ligands, Dll4 is expressed specifically in the endothelium at sites of vascular development and angiogenesis [24]. Dll4 is a regulator of luteal angiogenesis in the primate through the notch pathway [25]. Dll4 also regulates VEGF-mediated microvascular growth and branching by preventing excessive branching that leads to vascular dysfunction [26]. Fraser etc. have confirmed that blocking Dll4 in vivo in the primate ovary using an anti-Dll4 monoclonal antibody results in increased luteal angiogenesis and microvascular density [25], suggesting that Dll4 could be a target molecule for the therapy of ovarian angiogenesis.

We also found that constitutive Notch signalling in adult transgenic mice inhibits bFGF-induced angiogenesis and blocks ovarian follicle development. The ovaries of sterile IC-Notch 1 expressing females only had pre-antral and degenerative follicles but not antral follicles as seen in the ovaries from wildtype mice. Further, we have found that mice expressing IC-Notch1 had 30% less infiltration of ECs in bFGF matrigel plugs than wildtype mice and capillaries instead of larger vessels formed in IC-Notch1 expressing mice [27]. In marmosets, quantification of the area of CD31 staining revealed the extent of the microvascular tree within each corpus luteum was a significant increase on day 3 after anti-Dll4 treatments [25].

Moreover, compound E, a pan-Notch inhibitor, inhibits follicular development to the preovulatory stage with uninhibited vascular proliferation and disorganized appearance of ECs and VSMCs. Inactivation of Notch 1 ligand Dll4 on endothelial cells by blocking antibody YW152F leads to a mild disorganisation of follicular vasculature [23].

Based on the evidence above, we draw the conclusion that Notch pathway inhibits ovary angiogenesis and maintains the integrity of the ovarian vasculature (Table 1).

**Table 1:** The distinct role of targeting Dll4 in normal ovary and ovarian cancer

	Targeting Dll4	Ref.
	Blocking Dll4 in vivo in the primate ovary using an anti-Dll4 monoclonal antibody results in increased luteal angiogenesis and microvascular density.	[23]
Normal ovary	In marmosets, microvascular tree within each corpus luteum was a significant increase after anti-Dll4 treatments.	[23]
	Inactivation Dll4 with the blocking antibody YW152F induces a mild disorganisation of follicular vasculature.	[21]
Ovarian cancer	Dll4 RNAi silencing in ovarian tumour cells inhibited angiogenesis. Pericyte coverage was significantly decreased in the group treated with mouse Dll4 siRNA.	[31]

## Therapeutic targets to block Notch signalling pathway in patients with PCOS

As mentioned above, Notch signalling pathway is required for angiogenesis in normal ovary. As the ovaries from PCOS patients are characterized with abnormal angiogenesis, it is feasible to target Notch signalling pathway for blockade of abnormal ovary angiogenesis to treat PCOS. Several studies have demonstrated the potential targets of Notch signalling pathway in PCOS patients or animal models. Rat models of PCOS were induced by letrozole, and expressions of microRNAs were screened in PCOS rats and control rats. The researchers found that 129 miRNAs were differentially expressed in the ovaries from rat PCOS model compared with the control. Pathway analysis suggested that differentially expressed miR-201-5p, miR-34b-5p, miR-141-3p, and miR-200a-3p regulate oocyte meiosis, MAPK signalling, PI3K-Akt signalling, Rap1 signalling, and Notch signalling [28]. Further more, Bo Xu etc. have identified the altered miRNA expression profiles and miRNA targeted signalling pathways in 21 women with PCOS and 20 control women without the disease. They identified that 59 known miRNA were differentially expressed in PCOS cumulus granulosa cells. The Notch3 was demonstrated to be targeted by miR-483-5p based on quantitative real-time PCR, western blot and luciferase activity assay [29]. Ovarian angiogenesis and Notch target genes in PCOS could be of relevance for impaired oocyte competence [30]. Together; specific miRNAs regulating the Notch signalling pathway provides novel therapeutic targets for the treatment of PCOS.

## Targeting angiogenesis regulated by Notch pathway in ovarian cancers

### Notch signalling and angiogenesis in ovarian cancer

In addition to confirmation of the increased expression of Dll4, Notch 1, or Notch 3 in ovarian tumor tissues, Wang et al. have found that Dll4 was positively correlated with VEGFR1 expression, and Notch 1 was positively associated with VEGFR2 expression and microvessel density in the ovarian cancer tissues [31]. Using Affymetrix U133 plus 2.0 microarrays, Lu etc. have examined gene expression differences between endothelial cells from 10 invasive epithelial ovarian cancers and endothelial cells from 5 normal ovaries. Notch ligand Jagged 1 were over expressed in invasive epithelial ovarian cancers [32].

The over expressions of Dll4, Notch 1, Notch 3 or Jagged 1 suggest that Notch signalling plays a key role in angiogenesis in ovarian cancer. And the significant expression differences of Notch components between tumor and normal endothelium provide significant implications for the development of antiangiogenic therapies.

### Therapeutic inhibitors targeting Notch pathway in ovary tumor angiogenesis

Notch pathway inhibitors have been developed in recent years, and some inhibitors are in current clinical trials. The species of Notch inhibitors include monoclonal antibodies aiming to block Notch ligands/receptors, receptor decoys, gamma-secretase inhibitors (GSIs), and peptides aiming to block transcriptional complex. Gamma-secretase inhibitors are the most widely studied Notch pathway targeting agents which target all Notch receptors by preventing formation of the active NICD and it has been observed that GSIs has a promising anti-tumor role in several malignancies in early phase clinical trials. Richter S etc. have conducted the phase I study of an oral gamma secretase inhibitor RO4929097 in combination with gemcitabine in adult patients with advanced solid tumors and significant antitumor activity has been seen in several tumor types including pancreas and nasopharyngeal carcinoma. Of note, Notch protein expression was lower in patients who achieved prolonged stable disease or partial response [33]. Krop I etc. also have conducted the study of phase I pharmacologic and pharmacodynamic study of the gamma secretase inhibitor MK-0752 in adult patients with advanced solid tumors. In this study it has been observed that one patient had a confirmed complete response and 10 other patients had prolonged stable disease [34]. In addition, a phase II clinical trial of RO4929097 is ongoing in ovarian cancer patients (NCT01195343) [24], expecting that gamma-secretase inhibitors may have a similar role in inhibiting abnormal angiogenesis through Notch pathway during tumor angiogenesis as it have been shown in other types of tumors. However, gamma-secretase inhibitors broadly block all Notch signalling which may lead to side effects as Notch signaling also play a critical role in angiogenesis under physiological conditions, thus alternative and more specific methods of Notch pathway inhibition have been studied for antiangiogenic cancer therapy in ovarian cancer.

Lu etc. have demonstrated that silencing of Jagged 1 gene with a small interfering RNA blocked tube formation and migration of endothelial cells in vitro [32]. Adam D. Steg and colleagues also have confirmed that inhibition of Jagged 1 using Jagged 1 siRNA induced anti-angiogenic effects in ovarian tumor models. In their research, the ovarian cancer cell lines IGROV-AF1 and SKOV3Trip2 were used respectively and Jagged 1 silencing significantly decreased cell viability. Further, IGROV-AF1 and SKOV3Trip2 cell lines were used to generate intraperitoneal tumor in mice, and treatments with either mouse specific Jagged 1 siRNA, human specific Jagged 1 siRNA or both siRNA significantly reduced tumor growth, which is explained that microvessel densities in tumors were inhibited by anti Jagged 1 siRNA treatments, thus Jagged 1 inhibition therapy induced anti-angiogenic effects [35]. In addition to targeting Jagged 1 gene, Dll4-notch pathway is another therapeutic target for ovarian cancers. Wei Hu etc. have investigated the clinical and biological significance of Dll4 in ovarian cancer, they have observed that Dll4 was over expressed in 72% of tumors examined by IHC and confirmed that over expressed Dll4 was an independent predictor of poor survival when compared to samples with low Dll4 expression [36]. To further clarify the function of over expressed Dll4 in ovarian cancer, mice model harboring ovarian cancer cell lines A2780 or SKOV3ip1 derived xenografts were treated with Dll4 specific siRNA. And silencing Dll4 in tumor cells results in inhibition of tumor growth, consistently, silencing Dll4 in mouse tumor-associated endothelial cells significantly inhibits angiogenesis [36]. Moreover, given Dll4 plays an important role in pericyte formation during tumor vessel expansion [37], the extent of pericyte coverage were examined after Dll4 siRNA treatments. Pericyte coverage was significantly decreased in the group treated with mouse Dll4 siRNA in comparison to control. In addition to using Dll4 siRNA, monoclonal antibody is another option to block Dll4. Demcizumab (OMP-21 M18), monoclonal antibody targeting Dll4, is under phase Ib/II clinical development in ovarian cancer patients (NCT01952249) [24].

### Synergy effect on inhibiting ovarian tumor angiogenesis between Notch targeting and anti-angiogenic treatment

VEGF and Dll4-Notch pathways affect each other. VEGF increases Notch signalling components Dll4 expression in vivo and vitro. The expression of Dll4 in cultured endothelial cells was increased by VEGF treatments [38] (Fig. 2). Dll4 was strongly expressed on the front of growing vessels in vascularised tumors and Dll4 expression in tumor vessels was rapidly decreased after blocking VEGF [39]. In murine model of developing retina, blockade of VEGF significantly induced the decrease of sprouting and Dll4 expression on the vessels [22]. Dll4-Notch signalling can alter expressions of three VEGF receptors. It has been demonstrated that VEGFR1 expression may be increased by Notch signalling. The decrease of VEGFR1 expression was significantly induced in Dll4 heterozygous mice in which the Notch signaling activation was reduced [22]. In addition, activated Dll4-Notch signalling induced the increase of expressions of VEGFR1 and soluble VEGFR1 in cultured endothelial cells [40]. Conversely, Notch signalling can provide negative feedback to reduce the activity of the VEGF/VEGFR2 pathway. In vitro, it has been observed that VEGFR2 expression was decreased following activation of Notch in cultured endothelial cells [41]. In Dll4 heterozygous mice, it also has been demonstrated that VEGFR2 expression were increased in vessels [22]. VEGF induces Dll4 expression in tip cells, which in turn decreased VEGFR2 in stalk cells, thus the differentiation of tip and stalk cells were regulated differently in this way [42]. Notch signalling pathway could affect VEGFR3 expression through regulating VEGFR3 promoters. In addition, Notch signalling also alter VEGF responsiveness in human and murine endothelial cells through regulations of VEGF receptors expressions [43].

Hu and colleagues have found that combining Dll4-targeted siRNA with VEGF inhibition bevacizumab was more effective in inhibiting angiogenesis in preclinical models of cancer, and patients with tumors after treatment with anti-VEGF therapy had lower Dll4 expression [36]. Thus, targeting Dll4 in combination with VEGF inhibition potentially improves outcome of ovarian cancer treatments.

Moreover, there is a link between Notch signalling pathway and Nitric oxide/soluble guanylyl cyclase signalling. Nitric oxide (NO) produced by tumor, stromal and endothelial cells promotes proliferation and survival of ovarian cancer cells, mediating by soluble guanylyl cyclase (sGC). NO also promotes tumor angiogenesis at low concentrations, and it has been confirmed that Notch activation augments nitric oxide/soluble guanylyl cyclase signalling in immortalized ovarian surface epithelial cells and ovarian cancer cells [44], thus a combination of soluble guanylyl cyclase and Notch inhibition may also be a more effective combination in inhibiting angiogenesis in ovarian cancer.

## Conclusions

The ovary is an important organ to study angiogenesis and vascular function. In this review, we highlighted the expression pattern of Notch signalling pathway elements in the ovary and key roles for regulatory Notch signalling pathways on angiogenesis in pathological ovary. Notch signalling pathway is promising target for anti-angiogenesis therapy for treatment of ovarian diseases. A series of Notch pathway inhibitors have been developed, including monoclonal antibodies aiming to block Notch ligands/receptors, receptor decoys, gamma-secretase inhibitors, peptides aiming to block transcriptional complex or siRNA for Notch signalling pathway components. However, reports of the blockade of angiogenesis through Notch signalling pathway in PCOS has been limited, and it has been demonstrated that miRNAs targeting Notch signalling pathway are differentially expressed between PCOS and health controls. Notch signalling pathway regulates other processes in pathological ovary, such as progesterone secretion and the response to androgen. Women with PCOS usually have low progesterone levels which may be caused by the inhibitory role of Notch signalling on follicle-stimulating hormone (FSH)-induced expression of steroidogenic genes in granulosa cells of small preantral follicles [45]. Androgen excess in PCOS not only disturbs the balance between androgens, anti-Müllerian hormone (AMH) and FSH, but also contributes to ovarian tissue remodeling: stromal hyperplasia and rigidity, hypervascularity and inflammation [46] Androgen receptor are expressed in all cell types of the ovarian follicle, including the oocyte, granulosa and theca cells [47]. Hey1, a mediator of Notch signalling, is an androgen receptor corepressor [48] Thus, components of the Notch signalling pathway represent targets for regulating abnormal angiogenesis and other pathological process in ovary. Furthermore, the roles of Notch signalling pathway on anovulation, ovarian production of androgens and polycystic morphology, which are closely related to ovarian angiogenesis, have not fully been clarified. The underlying mechanisms need to be further explored in future.

## Abbreviations

AMH: Anti-Müllerian hormone
bFGF: Basic fibroblast growth factor
bHLH: Basic-helix-loop-helix
CL: Corpus luteum
EGF: Epidermal growth factor
FSH: Follicle-stimulating hormone
GSIs: Gamma-secretase inhibitors
HD: Heterodimerization domain
HDAC: Histone deacetylase
IC-Notch: Intracellular domain of Notch
LH: Luteinizing hormone
NLS: Nuclear localization sequences
NO: Nitric oxide
PCOS: Polycystic ovary syndrome
PDGF: Platelet-derived growth factor
PECAM: Platelet endothelial cell adhesion molecule
PEST: Proline, glutamate, serine, threonine rich
PlGF: Placental growth factor
sGC: Soluble guanylyl cyclase
SMRT: Silencing mediator of retinoid and thyroid hormone receptors
TAD: Transactivation domain
TD: Transmembrane domain
TGF-β: Transforming growth factor beta
VEGF: Vascular endothelial growth factor
VSMC: Vascular smooth muscle cell
